# Nipple Areolar Complex (NAC) Neurotization After Nipple-Sparing Mastectomy (NSM) in Implant-Based Breast Reconstruction: A Systematic Review of the Literature

**DOI:** 10.1155/tbj/2362697

**Published:** 2025-10-06

**Authors:** Thomas J. Sorenson, Carter J. Boyd, Jenn J. Park, Kshipra Hemal, Chris Amro, Nicholas Vernice, Alexis Lakatta, Oriana Cohen, Nolan Karp, Mihye Choi

**Affiliations:** Hansjorg Wyss Department of Plastic Surgery, NYU-Langone Health, New York, New York, USA

**Keywords:** implant-based breast reconstruction, neurotization, nipple neurotization, nipple-sparing mastectomy

## Abstract

**Background:**

Nipple-sparing mastectomy (NSM) with implant-based breast reconstruction (IBBR) preserves the nipple-areolar complex (NAC) with superior aesthetic results but results in loss of nipple sensation. Nipple neurotization has emerged as a technique to restore the sensory function, yet outcomes remain variable across studies. This systematic review synthesizes the available evidence on nipple neurotization in IBBR, focusing on sensory recovery, patient satisfaction, and surgical techniques.

**Methods:**

A systematic review was conducted following PRISMA guidelines. PubMed, Ovid EMBASE, and Cochrane Library were searched through April 1, 2025, for studies evaluating nipple neurotization in IBBR. Eligible studies included randomized controlled trials, cohort studies, and case series reporting surgical technique, sensory, and/or patient satisfaction outcomes. Data extraction included study characteristics, surgical techniques, sensory outcomes, and patient-reported satisfaction. Risk of bias was assessed using standardized tools.

**Results:**

Six studies met inclusion criteria, comprising 212 patients and 257 neurotized breasts. Sensory recovery was assessed using monofilament testing and patient-reported outcomes. Studies demonstrated overall improvement of NAC sensory outcomes and high patient satisfaction after neurotization. However, variability in neurotization methods, follow-up duration, and specific measured sensory outcomes limited direct comparisons.

**Conclusion:**

Nipple neurotization in IBBR shows promise in enhancing sensory recovery and patient satisfaction after NSM, but heterogeneity in surgical techniques and outcome measures, as well as poor study designs, limits definitive conclusions. Standardized protocols and randomized studies with long-term patient follow-up are needed to establish best practices and optimize neurotization outcomes.

## 1. Introduction

Nipple-sparing mastectomy (NSM) for breast cancer ablation has become increasingly popular due to its superior aesthetic outcomes and psychological benefits [1–5]. However, one notable limitation specific to NSM is loss of nipple-areolar complex (NAC) sensation, which can significantly impact patient satisfaction and quality of life [6, 7]. Traditional implant-based breast reconstruction (IBBR) prioritizes shape and symmetry but neglects the functional restoration of the NAC, including sensation. Nipple neurotization has gained attention as a promising adjunct to IBBR to restore sensation to the NAC following NSM [8, 9]. This technique aims to restore nipple sensation through a series of maneuvers that connect the transected lateral intercostal (IC) nerves with the underside of the NAC [10]. A variety of techniques with mixed results are reported in the literature. While some studies suggest that neurotization enhances sensory recovery, others highlight inconsistencies in outcomes and a lack of standardized techniques.

Despite the growing interest in nipple neurotization, there is no comprehensive synthesis of outcomes across different studies evaluating its efficacy in IBBR. Existing literature is fragmented, making it difficult for surgeons to determine best practices and for patients to have realistic expectations. This systematic review aims to analyze available evidence on nipple neurotization in IBBR, focusing on sensory recovery, patient-reported outcomes, and surgical techniques. By synthesizing current data, we seek to provide a clearer understanding of whether neurotization should become a standard component of IBBR and identify gaps for future research.

## 2. Methods

### 2.1. Study Design

This study is a systematic review conducted in accordance with the Preferred Reporting Items for Systematic Reviews and Meta-Analyses (PRISMA) guidelines and is registered with PROSPERO (ID# 1142637) [11].

### 2.2. Eligibility Criteria

Studies were included if they met the following criteria: (1) reported nipple neurotization patient outcomes in IBBR after NSM and (2) used any technique involving nerve coaptation, nerve grafting, or alternative neurotization methods. Exclusion criteria included studies discussing neurotization in autologous reconstruction, studies on non-neurotized NSM alone without a neurotization component, or studies with insufficient sensory outcome data or non-English publications without available translations.

### 2.3. Search Strategy

A comprehensive search of PubMed, Ovid EMBASE, and Cochrane Library was performed from database inception to April 1, 2025. A full list of search terms can be seen in Supporting Figure 1. The reference lists of included studies and relevant review articles were manually screened to identify additional eligible studies.

### 2.4. Study Selection

Two independent reviewers (TJS, JJP) screened all titles and abstracts. Full-text articles of potentially relevant studies were assessed for eligibility. Disagreements were resolved by consensus or a third reviewer (CJB).

### 2.5. Data Extraction and Synthesis

Data were extracted using a standardized form, including study characteristics (author, year, study design, sample size), patient demographics (age, BMI, comorbidities, prior radiation therapy), surgical technique (nerve type, coaptation method, grafting approach), outcome measures (sensory testing, patient-reported satisfaction, complications), and follow-up duration. Findings were synthesized descriptively. Primary outcomes included sensory recovery, and secondary outcomes included patient-reported satisfaction, complications, and aesthetic outcomes.

### 2.6. Risk of Bias and Quality Assessment

The risk of bias was assessed using Joanna Briggs Institute (JBI) Critical Appraisal Checklist for case series.

## 3. Results

### 3.1. Study Selection

**Figure 1:**
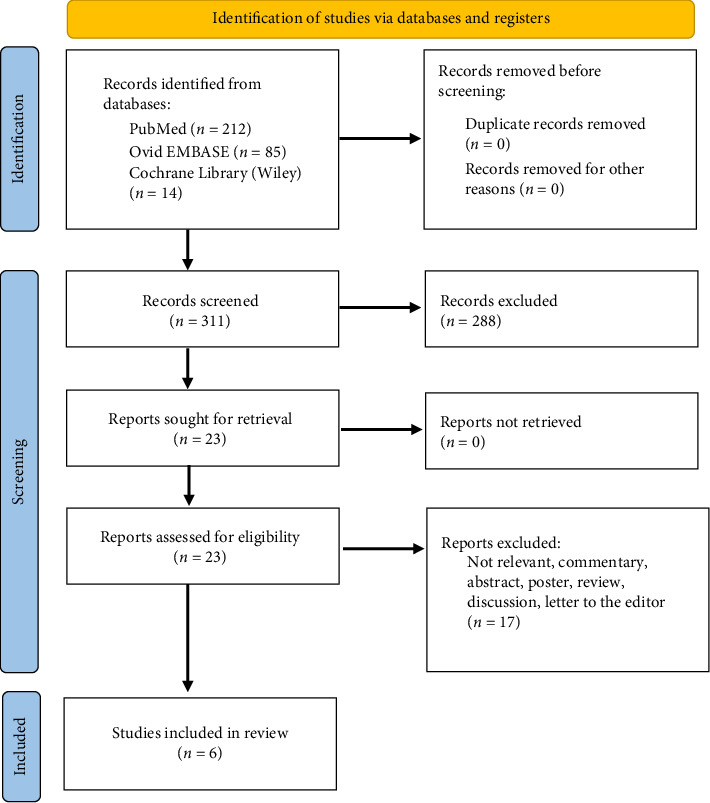
Flowsheet of included and excluded studies.

Our literature search identified 225 possible articles for inclusion. Six studies were included in the systematic review after screening (Figure 1).

### 3.2. Study Characteristics

There were six studies reporting on 212 patients included in the systematic review (Table 1) [10, 12–16]. Most (4/6; 66%) studies were retrospective case series. There were 305 total breasts included in this review, with 257 breasts undergoing neurotization. Two studies included non-neurotized breasts for comparison as well (*n* = 126) [15, 16]. Five studies reported the mean age of patients, which was overall 43 years. Four studies reported the mean BMI of patients, which collectively was 24 kg/m^2^. Three studies reported the incidence of prior radiation with two studies including no patients who underwent prior radiation, and one study including 22% of patients who underwent prior radiation. Three studies included the incidence of chemotherapy, with reported ranges from 19% to 39%.

### 3.3. Surgical Techniques and Procedural Variability

The neurotization method is variable among studies (Table 2). All authors use the lateral cutaneous branch of the third, fourth, or fifth IC nerve. In most cases, the nerves are transected as the donor nerve stump immediately as it emerges from the lateral chest wall without any dissection into the breast tissue. In some cases, when the distal nerves are easily identifiable coursing around the breast membrane or through breast peripheral parenchyma, they are dissected free using 3.5 loupe magnification for 2–3 cm, skeletonizing just around the epineurium only. No breast tissue is included in the dissected nerve. We do not dissect the nerve if the breast cancer is in proximity to the lateral chest wall and/or inferior outer quadrant of the breast. Four authors report using allograft to span the distance between the lateral cutaneous IC nerve stump and the NAC [10, 13, 14, 16]. Shyu et al. describe use of an IC nerve autograft [15]. All authors use neurorrhaphy to secure the distal IC nerve stump to the nerve graft. Two authors secure the coaptation with a nerve conduit [14, 16]. A variety of methods have been reported for the distal coaptation. Peled and Peled suture the graft to a previously identified subareolar nerve [10]. The remaining four authors attach the graft to the underside of the NAC in a targeted NAC reinnervation (TNR) style [17], a technique detailed extensively in numerous review papers [18, 19].

### 3.4. Sensory Recovery and Patient Satisfaction Outcomes

Objective sensory recovery outcomes were assessed in a variety of ways, including with Diskriminator (Sensory Management Services, LLC, Baltimore, Md.) [10], Acroval pressure-specified sensory device (PSSD) (Axogen, Jacksonville, Fla.) [12], Semmes-Weinstein monofilament test [14–16], and unique pressure-specific static and dynamic testing [13] (Table 3). Two studies mentioned assessing preoperative sensation [12, 14]. All studies divided the NAC into areolar quadrants for assessment, and studies had a variety of follow-up modalities that commenced assessment of sensation at approximately 3 months postoperatively and then about every 3 months thereafter. Specific follow-up times for each study are reported in Table 1. Two authors utilized the BREAST-Q questionnaire to assess patient-reported outcomes [14, 15], with one author utilizing an additional in-house institutional questionnaire [15].

In the Peled and Peled initial paper [10], NAC 2-point discrimination was found to be preserved compared with preoperative values in 87% of breasts in patients with at least 3 months of follow-up. The authors report reduced NAC sensation in 2 breasts (9%) and reported improvement in one breast (4%). All patients reported intact sensation to gross and light touch throughout the reconstructed breasts and good satisfaction with their sensory outcomes. Following their original publication, Peled et al. published a larger series of patients without the control group and reported that over 80% of patients had good-to-excellent one-point moving and one-point static sensibility scores averaged across all tested nipple quadrants at six- and 12-months follow-up. This patient cohort had an average of 9.2 months of follow-up [12], during which time no patients developed breast dysesthesias or clinical evidence of neuroma. Notably, this patient population was reported to have a very low rate of radiation (0%) and chemotherapy (< 20%).

Djohan et al. reported their group's evaluation of early sensation recovery in eight patients (15 breasts) [13]. Fifteen breasts underwent postoperative sensory testing at an average of four months after surgery, and five breasts underwent repeat testing at an average of 10 months after surgery (the remaining 10 breasts were lost to follow-up). They found that most of the breast skin and NAC surface area experienced sensory improvement when comparing the two postoperative time points. Further, two patients from their cohort underwent bilateral reconstruction and unilateral neurotization. In these two patients, neurotized breasts had better sensory testing thresholds in six of eight areas tested in comparison with non-neurotized breasts though exam was unblinded.

Using Semmes-Weinstein monofilament testing and the Breast-Q Breast Sensation Module to evaluate outcomes, Zhang et al. found that at 3 months postoperatively, sensation was impaired across all regions, but there was a significant improvement in sensation in all areas of the breast up to 12 months postoperatively, with the greatest improvement occurring between the six- and 12-month time interval [14]. On BREAST-Q, most patients reported improved sensation over time and no abnormal sensations at 12-month follow-up. Most recently, Shyu et al. published a prospective cohort study investigating neurotization in patients undergoing autologous and IBBR [15]. When investigating only IBBR (*n* = 42), seven (17%) patients were neurotized and 35 (83%) patients were not neurotized. They reported that, with a mean follow-up of over 1 year, most of the variables presented, including nipple sensation and satisfaction, trended toward better outcomes in patients with neurotization, but the differences did not reach statistical significance likely due to underpower. One exception was that patients reported significantly less itching in the neurotized cohort. The testing was not blinded.

Boyd et al. were the only group to investigate the costs associated with nipple neurotization in a matched-pair comparison study [16]. They found that neurotization added a mean cost of almost $8000 per breast. They also reported that rates of major and minor complications did not significantly differ between neurotized and non-neurotized groups.

### 3.5. Complications and Adverse Events

All included case studies reported on complications, including unplanned return to the operating room, implant infection requiring intravenous antibiotics or implant exchange/removal, wound dehiscence or skin flap/nipple necrosis requiring intervention, and seroma. Two authors provided comparative data for complications in neurotized and non-neurotized breasts [15, 16]. Reported complication rates were all under 10% (Table 1). No included patients were reported to have complications related to the nerve surgery (neuroma formation, chronic nerve pain, allodynia) during the follow-up periods. No studies reported data on oncologic outcomes after NAC neurotization.

### 3.6. Summary of Findings and Evidence Quality

Overall, the early published results of NAC neurotization after NSM are promising, with early evidence that the neurotization procedure is effective with rare surgical complications and no reported neurotization-related complications. Patient satisfaction, as measured by standardized questionnaires, is high, and nipple sensation, as measured by objective testing, appears to improve after the neurotization procedure. There is a non-negligible risk of bias as all included studies are small case series, so the risk of bias was assessed using the JBI Critical Appraisal Checklist [20]. Three (3/6; 50%) of the included studies were written by authors with related financial interests [10, 12, 13], which has been found to be very common [21].

## 4. Discussion

Six studies met inclusion criteria, comprising 212 patients and 257 neurotized breasts. Studies demonstrated overall improvement of NAC sensory outcomes and high patient satisfaction after neurotization. Operative techniques were variable between authors with some similarities. In early, short-term follow-up data, results appear promising, though variability in methods of measuring and tracking sensory outcomes limits direct comparisons.

NSM is the aesthetic gold standard and has been found to be oncologically safe to perform for both prophylactic and therapeutic mastectomy in select patients, but has functional limitations, including reported loss of nipple sensation [4, 5, 22–25]. A large study of 460 patients undergoing NSM from Lai et al. found a significant portion of patients with decreased or absent nipple sensation after NSM [26]. These results would suggest that there is a real need for surgical innovation in this space to improve sensation outcomes and patient satisfaction after their reconstruction. In early, short-term heterogenous data synthesized herein, NAC neurotization by reconstructing a lateral anterior IC nerve branch to the underside of the NAC appears to be that surgical innovation for the NSM patient who desires improved NAC sensation postoperatively. Nevertheless, there is a paucity of long-term and true comparative data between neurotized and non-neurotized cohorts regarding sensory outcomes. Thus, definitive conclusions regarding this technique are premature. Further, there is no standardized, widely accepted approach to NAC neurotization and postoperative sensory testing, which makes comparison between surgeons challenging. Additionally, there is evidence that NAC sensation improves even with no nerve reconstruction, so the necessity of neurotization remains uncertain, particularly given the increased costs of the procedure [23, 27–29]. Separately, robotic mastectomy has been shown to have improved sensibility following mastectomy compared to traditional techniques highlighting another area of investigation when seeking to optimize sensory outcomes [30]. Despite these shortcomings, BREAST-Q data reports strong patient satisfaction with this technique.

The findings of this systematic review provide important implications for both patients and surgeons. For patients, this represents a new frontier to address established dissatisfaction with sensory outcomes and an opportunity to improve overall quality of life. For surgeons, it is important to recognize that no data are available to determine the long-term efficacy and restoration of sensory outcomes, nor the oncologic outcomes of performing this procedure. Until studies report patient sensory outcomes with adequate follow up, patients should be informed that nipple neurotization in IBBR is experimental and any potential benefits of neurotization must be weighed against the primary concern of oncologic safety. Despite this, many will continue to offer this procedure, but patients should be appropriately counseled of the indeterminate nature of outcomes and critical attention should be paid to oncologic outcomes in these neurotization patients. Furthermore, the limitations in the literature should serve as a call to action for surgeons to design and implement high-quality studies and generate useful data for truly determining the efficacy of this technique in providing superior sensory outcomes without increasing oncologic risks. Regarding the ultimate outcomes, non-inferiority of nipple neurotization is not enough, given the high additional cost accrued in performing the procedure. With well-designed studies, nipple neurotization could very well become a standard practice in IBBR. However, the current literature is too heterogenous with insufficient follow-up to draw any definitive conclusions.

There are limitations to this systematic review. The primary studies included in our review did not explicitly frame their research questions using the PICO methodology. Rather, most were retrospective cohort or small case series designs that reported on neurotization techniques and subsequent sensory outcomes, often without a defined comparator group. As such, while we were able to interpret the findings within a PICO framework as best as possible, most original studies themselves did not prospectively employ PICO methodology and are subject to selection and reporting bias. Testing is largely nonblinded which introduces reporting bias. The reported follow-up duration is short relative to the time duration of nerve regeneration, and there is a wide range of surgeon and technique variability. Further, the included data do not represent the normal milieu of patients normally seen at a comprehensive cancer or university hospital, which limits external validity. Lastly, as the authors of these studies often have financial disclosures relevant to the future success of NAC neurotization, there is a strong risk for confirmation bias.

Further research on this topic is essential. There is early data suggestive of the potential benefit of the nipple neurotizaiton; however, a prospective, randomized, single-blinded study with standardized surgical technique, sensory outcome assessment, and long-term follow-up should be conducted. Evaluation of novel nerve coaptation technologies, like Nerve Tape (Biocircuit Technologies, Atlanta, GA, USA), would also be useful to identify potential refinements of the surgical technique that could help improve outcomes and reduce the length of the procedures.

## 5. Conclusions

Early data on nipple neurotization after NSM in IBBR suggest mild improvements in restoring sensory recovery and patient satisfaction. Heterogeneity in surgical techniques and outcome measures, as well as inadequate study designs, limits definitive conclusions that can be drawn. Standardized protocols and randomized studies with long-term patient follow-up are needed to establish best practices and optimize neurotization outcomes.

## Figures and Tables

**Table 1:** Summary of included studies.

Authors (year)	Study type	Total patients	Total breasts		Radiation	Chemotherapy	Follow-up duration	Major complication rate
Peled (2019)	Case series	16	Neurotized	31	*NR*	*NR*	12 patients with 3 months 6 patients with 6 months	NR
			Non-neurotized	0				

Djohan (2020)	Case series	8	Neurotized	15	0 (0%)	3 (3/8; 38%)	Mean: 10.59 months (±3.57)	0 (0%)
			Non-neurotized	0				

Peled (2023)	Case series	47	Neurotized	79	0 (0%)	9 (9/47: 19%)	Mean: 9.2 months (range: 6–14)	2 (2/79; 2.5%)
			Non-neurotized	0				

Zhang (2024)	Case series	54	Neurotized	92	12 (12/96; 13%)	21 (21/54; 39%)	31 patients with 6 months 27 patients with 12 months	4 (4/54; 7.4%)
			Non-neurotized	4				

Shyu (2025)	Prospective cohort study	42	Neurotized	14	3 (21%)	*NR*	Mean: 1.3 years (±0.4)	“No statistically significant differences were noted in…surgical complications”
			Non-neurotized	70	5 (7.1%)		Mean: 1.3 years (±0.3)	

Boyd (2024)	Matched-pair comparison	45	Neurotized	26	*NR*	*NR*	Mean: 129.2 days	1 (1/26; 3.9%)
			Non-neurotized	52			Mean: 417.3 days	6 (6/52; 11.5%)

Abbreviation: NR, not reported.

**Table 2:** Summary of surgical techniques.

Authors (year)	Donor nerve	Type of nerve graft	Mode of proximal coaptation	Mode of distal coaptation
Peled (2019)	Lateral cutaneous branch of 3^rd^, 4^th^, or 5^th^ IC nerve	Allograft	Neurorrhaphy	Neurorrhaphy to subareolar nerve
Djohan (2020)	Lateral cutaneous branch of 4^th^ IC nerve	Allograft	Neurorrhaphy	Attach to underside of NAC (TNR style)
Peled (2023)	Lateral cutaneous branch of 3^rd^, 4^th^, or 5^th^ IC nerve	Allograft	Neurorrhaphy	Neurorrhaphy to subareolar nerve
Zhang (2024)	Lateral cutaneous branch of 3^rd^, 4^th^, or 5^th^ IC nerve	Allograft	Neurorrhaphy with nerve conduit	Attach to underside of NAC (TNR style)
Shyu (2025)	Lateral cutaneous branch of 3^rd^, 4^th^, or 5^th^ IC nerve	IC nerve autograft	Neurorrhaphy	Attach to underside of NAC (TNR style)
Boyd (2025)	Lateral cutaneous branch of 3^rd^, 4^th^, or 5^th^ IC nerve	Allograft	Neurorrhaphy with nerve conduit	Neurorrhaphy with nerve conduit

*Note:* IC, intercostal.

Abbreviations: NAC, nipple areolar complex; TNR, target NAC reinnervation.

**Table 3:** Summary of methods of sensory and patient satisfaction outcomes.

Authors (year)	Method of assessing sensory outcomes	Method for assessing patient satisfaction outcomes
Peled (2019)	Diskriminator	NR
Djohan (2020)	Unique pressure specific static and dynamic testing	NR
Peled (2023)	Acroval pressure-specific sensory device (PSSD)	NR
Zhang (2024)	Semmes–Weinstein monofilament test	BREAST-Q
Shyu (2025)	Semmes–Weinstein monofilament test	BREAST-Q + an additional institutional questionnaire
Boyd (2025)	Semmes–Weinstein monofilament test	NR

Abbreviation: NR, not recorded.

## Data Availability

The data that support the findings of this study are available from the corresponding author upon reasonable request.

## Supporting Information

Supporting Figure 1. Search strategy.
