# Supplementary material for: Nipple Areolar Complex (NAC) Neurotization After Nipple-Sparing Mastectomy (NSM) in Implant-Based Breast Reconstruction: A Systematic Review of the Literature
Source: Breast J. 2025 Oct 6;2025:2362697. doi: 10.1155/tbj/2362697 (PMC12517998; doi:10.1155/tbj/2362697)
Supplement: Supporting Information — Additional supporting information can be found online in the Supporting Information section. [file 2362697.f1.docx]

**PubMed** (ALL – 1946 to March 15, 2025) – **212 results**

Searched on March 15, 2025

No language, article type, or publication date restrictions

| **Line #** | **Search** |
| --- | --- |
| 1 | (nipple areolar complex OR NAC) |
| 2 | AND |
| 3 | (reinnervation OR innervation) |

**Ovid EMBASE** (1974 – Present) – **85 results**

Searched on March 15, 2025

No language, article type, or publication date restrictions

| **Line #** | **Search** |
| --- | --- |
| 1 | (breast* OR (breast* AND (nipple* OR NAC* OR nipple sparing*)) OR mastectomy*) |
| 2 | AND |
| 3 | (reinnervation* OR innervation* OR neurotization*) |

**Cochrane Library (Wiley) – 14 results**

Searched on March 15, 2025

No language, article type, or publication date restrictions

| **Line #** | **Search** |
| --- | --- |
| 1 | Mastectomy |
| 2 | AND |
| 3 | Neurotization OR innervation |
